# Olfactory and gustatory dysfunctions of COVID‐19 patients in China: A multicenter study

**DOI:** 10.1002/wjo2.5

**Published:** 2022-03-22

**Authors:** Jianhui Li, Yi Sun, Enqiang Qin, Hu Yuan, Mingbo Liu, Wenqi Yi, Zhu Chen, Chengcheng Huang, Fengjie Zhou, Ruiyao Chen, Leibo Zhang, Ning Yu, Qiong Liu, Xuejun Zhou, Jingjing He, Boyu Li, Fusheng Wang, Changliang Yang, Shiming Yang

**Affiliations:** ^1^ College of Otolaryngology Head and Neck Surgery Chinese PLA General Hospital Beijing China; ^2^ National Clinical Research Center for Otolaryngologic Diseases Beijing China; ^3^ Department of Otolaryngology Head and Neck Surgery Chinese PLA General Hospital Hainan Hospital Sanya China; ^4^ Hainan Clinical Research Center for Otolaryngologic Diseases Sanya China; ^5^ Department of Otolaryngology Head and Neck Surgery General Hospital of Central Theater Command Wuhan China; ^6^ Treatment and Research Center for Infectious Diseases The Fifth Medical Center of Chinese PLA General Hospital Beijing China; ^7^ National Clinical Research Center for Infectious Diseases Beijing China

**Keywords:** COVID‐19, gustatory, olfactory, SARS‐CoV‐2

## Abstract

**Introduction:**

With the spread of the epidemic worldwide, an increasing number of doctors abroad have observed the following atypical symptoms of coronavirus disease 2019 (COVID‐19): olfactory or taste disorders. Therefore, clarifying the incidence and clinical characteristics of olfactory and taste disorders in Chinese COVID‐19 patients is of great significance and urgency.

**Materials and Methods:**

A retrospective study was conducted, which included 229 severe acute respiratory syndrome coronavirus 2 confirmed patients, through face‐to‐face interviews and telephone follow‐up. Following the completion of questionnaires, the patients participating in the study, were categorized according to the degree of olfactory and taste disorders experienced, and the proportion of each clinical type of patient with olfactory and taste disorders and the time when symptoms appeared were recorded.

**Results:**

Among the 229 patients, 31 (13.54%) had olfactory dysfunction, and 44 (19.21%) had gustatory dysfunction. For the patients with olfactory dysfunction, 6 (19.35%) developed severe disease and became critically ill. Olfactory dysfunction appeared before the other symptoms in 21.43% of cases. The proportion of females with olfactory and gustatory dysfunction was higher than that of males (*P* < 0.001).

**Conclusions:**

The incidence of olfactory and gustatory dysfunction was much lower than that reported abroad; the prognosis of patients with olfactory dysfunction is relatively favorable; olfactory and gustatory dysfunction can be used as a sign for early screening; females are more prone to olfactory and gustatory dysfunction.

## Highlights

- It clarify the incidence and clinical characteristics of olfactory and gustatory dysfunction in Chinese COVID‐19 patients.
- This investigation compensates for the previous lack of data on olfactory and gustatory dysfunction in Chinese COVID‐19 patients.
- It provides important clues and evidence for the current screening of patients, especially targeting asymptomatic carriers and worldwide epidemic prevention and control.

## INTRODUCTION

According to reports, since the outbreak of the coronavirus disease 2019 (COVID‐19) epidemic at the end of 2019, the most common clinical manifestations of patients in China are fever, cough, dyspnea, fatigue, and loss of appetite.^1^ With the spread of the epidemic around the world, an increasing number of doctors abroad have observed the following atypical symptoms of COVID‐19: olfactory or gustatory dysfunction.^2^ Compared with COVID‐19 patients in China, it remains unclear whether the changes in the clinical manifestations abroad are due to differences in pathogenic viruses. Therefore, it is of great significance and urgency to clarify the incidence and clinical characteristics of olfactory and gustatory dysfunction in Chinese COVID‐19 patients. We conducted a follow‐up survey of 229 patients with COVID‐19, obtained the incidence of olfactory and gustatory dysfunction in these patients and analyzed their clinical characteristics. This investigation compensates for the previous lack of data on olfactory and gustatory dysfunction in Chinese COVID‐19 patients. It provides important clues and evidence for the current screening of patients, especially targeting asymptomatic carriers and worldwide epidemic prevention and control.

## MATERIALS AND METHODS

Based on the positive results of the severe acute respiratory syndrome coronavirus 2 (SARS‐CoV‐2) viral nucleic acid test, a cross‐sectional study was conducted that included 229 COVID‐19 patients who were admitted to the Fifth Medical Center of the PLA General Hospital and General Hospital of Central Theater Command from December 2019 to January 2020. The following inclusion criteria were considered: laboratory‐confirmed COVID‐19 infection (reverse transcription‐polymerase chain reaction) and clinical ability to complete the questionnaire. The following exclusion criteria were considered: patients unable to independently complete the questionnaire survey; patients with previous olfactory or gustatory dysfunction; and patients who were currently in the intensive care unit). The severity of COVID‐19 was defined according to the diagnostic and treatment guidelines for SARS‐CoV‐2 issued by the Chinese National Health Committee (version 3–5). Severe COVID‐19 was designated when the patients had one of the following criteria: respiratory distress with respiratory frequency ≥30/min; pulse oximeter oxygen saturation ≤93% at rest; and oxygenation index (artery partial pressure of oxygen/inspired oxygen fraction, PaO_2_/FiO_2_) ≤300 mmHg. Demographic analysis, clinical characteristics, and laboratory test results were obtained from the patients' medical records. The questionnaire, which consisted of five general questions (age, sex, date of diagnosis, comorbidities, symptoms associated with COVID‐19 infection) and 10 questions about olfactory and gustatory function, was designed and carried out by an otolaryngologist and an infectious disease doctor. The visual analog scale was used to score the impairment of olfactory and gustatory function, where a higher the score indicated more serious symptoms. Patients in the hospital completed the questionnaire through face‐to‐face interviews. Patients who had been discharged completed the questionnaire by telephone. The data were also collected through an online form for discharged patients. Patients were classified according to the degree of olfactory disturbance, and the proportion of each clinical type in each degree was calculated. A statistical package (SPSS version 22.0) was used to perform the statistical analyses. The data collection time was from March 26, 2020 to April 15, 2020.

## RESULTS

A total of 229 patients diagnosed with COVID‐19 were enrolled in this study, including 119 males and 110 females. Their ages ranged from 13 to 90 years old, with an average age of 51.97 years old. Of the 229 patients, 218 showed symptoms related to COVID‐19, including fever, cough, dyspnea, fatigue, and loss of appetite; 55 had hypertension, 46 had other cardiovascular and cerebrovascular diseases, 34 had chronic gastritis, 29 had diabetes, 27 had chronic lung disease, 15 had chronic nephritis, and 8 had hepatitis B. There were 31 cases of olfactory dysfunction, with 8 (25.81%) cases of complete loss, 13 (41.94%) cases of moderate to severe decline, and 10 (32.26%) cases of mild decline (Table 1). There were 44 cases of gustatory dysfunction, with 2 (4.55%) cases of complete loss, 36 (81.82%) cases of moderate to severe decline, and 6 (13.64%) cases of mild decline. Among the patients who developed olfactory dysfunction, only 6 (19.35%) developed the severe disease, and 25 (80.65%) developed the ordinary disease (Table 1). Twenty‐eight of the Thirty‐one patients with olfactory dysfunction clearly described a fluctuation in their symptoms. Six (21.43%) cases occurred before other symptoms appeared, and the average number of days in advance of other symptoms was 3.5, which was a negative value; in 3 (10.71%) cases, olfactory symptoms occurred at the same time as other symptoms; in 19 (67.86%) cases, they occurred after other symptoms appeared, and the average number of days of delay was 4.89, which was a positive value. In general, 28 patients developed olfactory dysfunction at an average of 3.75 days after onset (Table 2). Of the 31 patients with olfactory dysfunctions, 21 (67.74%) were females, and 10 were males (32.26%). Of the 44 patients with gustatory dysfunction, 26 (59.09%) were females, and 18 were males (40.91%). Females were more likely to suffer from olfactory and gustatory dysfunction than males (*P* < 0.001).

**Table 1:** The proportion of different clinical types of 31 patients according to a different degree of olfactory dysfunctions (*n* [%])

Clinical type	Anosmia	Moderate olfactory dysfunctions	Mild olfactory dysfunctions	Total
Severe	0 (0)	4 (12.90)	2 (6.45)	6 (19.35)
Ordinary	8 (25.81)	9 (29.03)	8 (25.81)	25 (80.65)
Total	8 (25.81)	13 (41.94)	10 (32.26)	31 (100.00)

**Table 2:** The occurrence time about olfactory dysfunctions of 28 patients

Item	Time of olfactory dysfunctions			Total
	Before other symptom	At the same time	After other symptom	
Number of people (%)	6 (21.43)	3 (10.71)	19 (67.86)	28 (100.00)
Average number of days	−3.5	0	4.89	3.75

## DISCUSSION

COVID‐19 is a novel infectious disease, and its pathogenesis and pathophysiology remain unclear. Studies have shown that coronavirus infection is associated with olfactory impairment, but the mechanism by which SARS‐CoV‐2 virus infection causes olfactory and gustatory dysfunctions is still unclear.^3^, ^4^ The incidence of olfactory dysfunction and that of gustatory dysfunction in Chinese COVID‐19 patients are 13.54% and 19.21%, respectively; these findings agree with other authors' reports from China but are much lower than the reported rates of 85.6% and 88.8% in foreign countries.^5^, ^6^ The possible reason is that the virus has mutated during the transmission process, or there are different strains. Genetic and epidemiological data suggest that the D614G spike variant may be, in part, responsible for the increased frequency of chemosensory dysfunction during the current pandemic.^7^ Different subtypes of viruses have different affinities and neurotoxicity to mucosal receptors in the olfactory and gustatory regions, resulting in differences in their biological behavior.^8^ In addition, recent studies have shown that the expression of angiotensin‐converting enzyme 2, the receptor of SARS‐CoV‐2, differs in populations and tissues. ^7^ Therefore, different populations have different susceptibilities to SARS‐CoV‐2 and different clinical manifestations. In addition, the outcomes are also different.^9^

Among the patients with olfactory dysfunction, the proportion of patients who developed severe and critical illness was relatively small, that is, only 6 (19.35%) cases. This indicates that patients with olfactory dysfunction have a relatively good prognosis. The possible mechanism is that the virus in these patients is mainly concentrated in the upper airway, especially the nasal cavity and pharyngeal cavity. Only a tiny amount of virus develops downward into the lower airway or other tissues and organs to cause‐related clinical symptoms. As a gateway for humans to communicate with the outside world, a virus attack on the nasal or pharyngeal cavity may trigger a series of early warning mechanisms to quickly mobilize the body's immune system to combat the virus attack, thus preventing the virus from further attacking other vital tissues and organs. University of California San Diego health researchers published online research results in the International Forum of Allergy and Rhinology on April 24, 2020 and pointed out that loss of olfaction may predict a milder clinical course of COVID‐19. Researchers believe that these findings may suggest that some of the pathophysiological characteristics of this infection, the location and dose of the initial viral load, and the effectiveness of the host's immune response are potentially essential variables that determine the spread of the virus in the human body and, ultimately, the clinical course of infection.^10^ Loss of smell may also mean that a robust immune response occurred in the nasal passages while reactions in other parts of the body are limited.

Olfactory dysfunction occurred on average 3.75 days after the onset of the disease, slightly different from the 4.4 days reported abroad.^11^ It is worth noting that of the 31 patients with olfactory dysfunction, in 6 (21.43%) cases, olfactory dysfunction occurred before other symptoms appeared. Foreign patients who report olfactory disturbance as the first symptom account for up to 26.6% of the total COVID‐19 patients.^12^ Domestic scholars also found that apart from cerebrovascular disease and impaired consciousness, most neurologic manifestations occurred early in the illness (median time, 1–2 days).^6^ Therefore, using olfactory or gustatory dysfunction as an early warning signal and screening indicator will provide simple, accurate, and effective detection for large‐scale population screening. Early identification of suspected patients, isolation monitoring, and early diagnosis and treatment of COVID‐19 patients are of great significance for effectively controlling the spread of the epidemic and reducing the incidence of severe illness.

Among patients with olfactory dysfunction, females accounted for 67.74%, significantly higher than males. The possible reason may be the difference in the inflammatory response process between sexes.^13^ In addition, due to the effect of estrogen, women have a more sensitive sense of smell than men, and the fluctuations in the sense of smell for women are more obvious than those for men and are more susceptible to external factors. The olfactory epithelium is one of the target organs of estrogen. Primary olfactory sensory cells contain estrogen metabolic enzymes, which affect the effect of estrogen on the olfactory epithelium.^14^

There are still some limitations in this study. First, since some of the patients had been discharged from the hospital after recovery and some of the patients with hyposmia had recovered their sense of smell by the time we conducted the survey, we did not perform smell tests on the patients but used on‐site inquiries or telephone follow‐up questionnaires and visual analog scales to assess the olfactory status of the patients during the illness.^15^, ^16^ There was a moderate correlation between ratings and measures of olfactory function.^17^ The combined olfactory score correlated with the visual analog score, and the correlation coefficient was 0.56 (*P* < 0.01).^18^ Second, it is difficult for some patients to give a clear answer to the exact time and duration of olfactory and gustatory dysfunction. In addition, this study was a cross‐sectional study from March 26, 2020, to April 15, 2020. According to our short‐term follow‐up, most of the patients with smell and taste disorders returned to normal or basically normal function with the improvement of their condition. The lack of long‐term follow‐up of patients makes it impossible for us to know whether patients who had not recovered their sense of olfactory and gustatory function will recover their functions in the future and whether patients who are currently recovering will relapse. Last, only 214 patients were studied, and the sample size in some groups was small, which could cause biases in clinical observation. Next, we will increase the sample size to obtain more reliable conclusions.

## CONFLICT OF INTERESTS

The authors declare that there are no conflict of interests.

## ETHICS STATEMENT

This study is a retrospective study based on clinical data. The Medical Ethics Committee of PLA General Hospital has confirmed that no ethical approval is required.

## AUTHOR CONTRIBUTIONS

All authors contributed to the study conception and design. Shiming Yang, Changliang Yang, and Fusheng Wang supervized the overall study. Yi Sun, Enqiang Qin, Chengcheng Huang, Fengjie Zhou, Ruiyao Chen, Leibo Zhang, Zhu Chen, and Boyu Li collected clinical data. Jianhui Li and Wenqi Yi made the table. Hu Yuan, Qiong Liu, Jingjing He, and Xuejun Zhou searched the literature. The first draft of the manuscript was written by Jianhui Li, Qiong Liu, Mingbo Liu, and Wenqi Yi, and all authors commented on previous versions of the manuscript. All authors read and approved the final manuscript.

## Data Availability

Data and material are available.
